# PPARα Modulation-Based Therapy in Central Nervous System Diseases

**DOI:** 10.3390/life11111168

**Published:** 2021-11-02

**Authors:** Deokho Lee, Yohei Tomita, William Allen, Kazuo Tsubota, Kazuno Negishi, Toshihide Kurihara

**Affiliations:** 1 Laboratory of Photobiology, Keio University School of Medicine, Tokyo 160-8582, Japan; deokholee@keio.jp; 2 Department of Ophthalmology, Keio University School of Medicine, Tokyo 160-8582, Japan; kazunonegishi@keio.jp; 3 Boston Children’s Hospital, Harvard Medical School, Boston, MA 02115, USA; william.allen@childrens.harvard.edu; 4 Tsubota Laboratory, Inc., Tokyo 160-0016, Japan; tsubota@tsubota-lab.com

**Keywords:** central nervous system, eye, peroxisome proliferator-activated receptors

## Abstract

The burden of neurodegenerative diseases in the central nervous system (CNS) is increasing globally. There are various risk factors for the development and progression of CNS diseases, such as inflammatory responses and metabolic derangements. Thus, curing CNS diseases requires the modulation of damaging signaling pathways through a multitude of mechanisms. Peroxisome proliferator-activated receptors (PPARs) are a family of nuclear hormone receptors (PPARα, PPARβ/δ, and PPARγ), and they work as master sensors and modulators of cellular metabolism. In this regard, PPARs have recently been suggested as promising therapeutic targets for suppressing the development of CNS diseases and their progressions. While the therapeutic role of PPARγ modulation in CNS diseases has been well reviewed, the role of PPARα modulation in these diseases has not been comprehensively summarized. The current review focuses on the therapeutic roles of PPARα modulation in CNS diseases, including those affecting the brain, spinal cord, and eye, with recent advances. Our review will enable more comprehensive therapeutic approaches to modulate PPARα for the prevention of and protection from various CNS diseases.

## 1. Introduction

**Figure 1:**
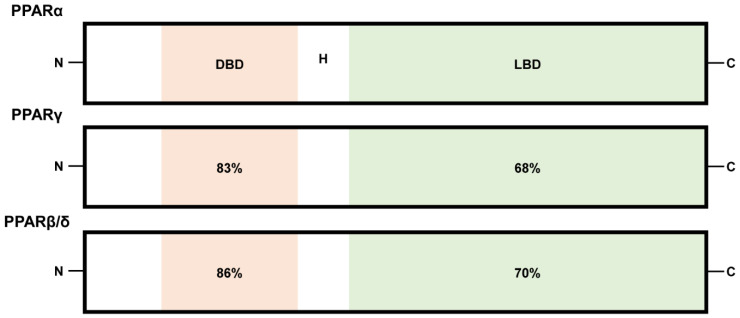
A schematic illustration of functional domains of PPARs (PPARα, PPARβ/δ, and PPARγ). N and C represent N-terminus and C-terminus, respectively. DBD and LBD represent DNA-binding domain and ligand-binding domain, respectively. H represents a hinge region. Numbers: percentages (%) identical to human PPARα.

Peroxisome proliferator-activated receptors (PPARs) belong to the family of ligand-regulated nuclear receptors, including PPARα, PPARβ/δ, and PPARγ. These receptors bind to DNA as heterodimers with retinoid X receptors (RXRs) and act as transcription factors to activate PPAR-inducible gene expression processes [1]. PPARs are encoded by distinct genes (PPARα, NR1C1; PPARβ/δ, NUC1 or NR1C2; PPARγ, NR1C3), which are located on chromosomes 15, 17, and 6 in mice and chromosomes 22, 6, and 3 in humans [2,3]. Structural and functional analyses demonstrated that the N-terminal DNA-binding domains (DBD) of PPARα, PPARβ/δ, and PPARγ are about 80% identical, while the C-terminal ligand-binding domains (LBD) separated by a hinge region (H) show approximately 60 to 70% identity (Figure 1) [4,5]. Polyunsaturated fatty acids are considered as preferred endogenous PPAR ligands [6,7,8,9]. Furthermore, various lipids such as saturated fatty acids, fatty acyl-CoA species, prostaglandins, leukotrienes, oxidized fatty acids, and oxidized phospholipids have been considered PPAR activators [6,7,8,9]. The investigation of physiologically relevant endogenous ligands for PPARs continues [10].

**Figure 2:**
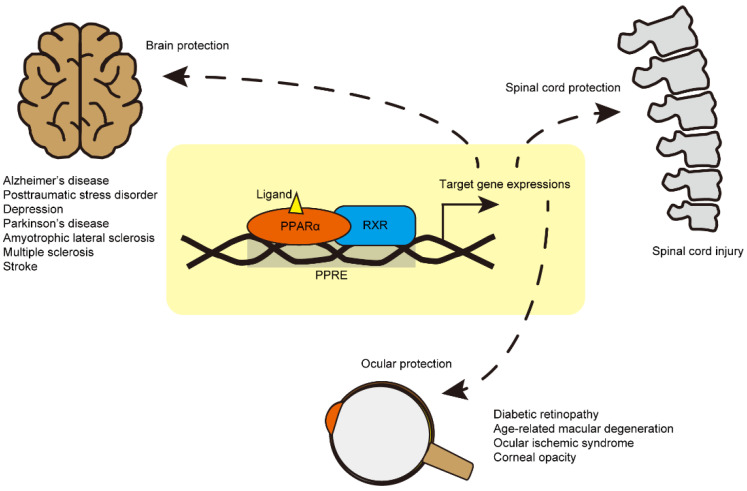
A schematic view of PPARα modulation therapy in central nervous system (CNS) diseases. The PPARα-retinoid X receptor (RXR) heterodimer binds to the peroxisome proliferator response element (PPRE; AGGTCANAGGTCA with unknown redundancy) in the nucleus (yellow box). It induces expressions in a variety of PPARα target genes, which are involved in anti-inflammation, protection, and the metabolism of glucose and lipid. To date, therapeutic roles of PPARα activation by PPARα agonist (ligand, yellow triangle) have been suggested in brain diseases (Alzheimer’s disease: AD, post-traumatic stress disorder: PTSD, depression, Parkinson’s disease: PD, amyotrophic lateral sclerosis: ALS, multiple sclerosis: MS, and ischemic stroke), spinal cord injury, and eye diseases (diabetic retinopathy: DR, age-related macular degeneration: AMD, ocular ischemic syndrome: OIS, and corneal opacity).

PPARα is mainly expressed in the liver, where it controls the oxidation of fatty acids and regulates lipoprotein metabolism [11,12]. PPAR-δ/β is involved in the modulation of macrophage-derived inflammation and fatty acid metabolism [11,12]. PPARγ is mainly expressed in white and brown adipose tissues and regulates insulin sensitivity [11,12]. Furthermore, PPARγ exerts various roles in regulating the development, metabolism, and inflammatory responses of the central nervous system (CNS) [12,13]. In this regard, the therapeutic role of PPARγ modulation in CNS diseases has been heavily researched and well reviewed [13]. To date, PPARγ has been a focal point in the modulation of neuro-inflammation for Alzheimer’s disease (AD) [14]. However, recently, PPARα modulation has emerged as a novel therapeutic target in various brain, spinal cord, and eye diseases. As such, the role of PPARα modulation in CNS diseases must be collated [15,16,17]. In this regard, we focused on reviewing the therapeutic effects of PPARα modulation as a promising approach for the treatment of various CNS diseases (Figure 2).

## 2. Brain Diseases

The distribution of PPAR isotypes in mouse and human brains has been recently studied even though therapeutic effects of PPARs have been examined in various brain diseases [18]. In the prefrontal cortex and nucleus accumbens of the adult mouse brain, PPARα expression was significantly greater than PPARγ expression [18]. In other subregions (the amygdala and ventral tegmental area), there was no significant difference between PPARα and PPARγ [18]. Furthermore, PPARα was detected as the only isotype to colocalize with all types of cells (neurons, astrocytes, and microglia) in both adult mouse and adult human brain tissues, which implies that PPARα may have extensive roles depending on the cell type in human and mouse brains [18].

In the disease state of AD, PPARα is a potential therapeutic target. The knockdown of PPARα reduced the expression of the α-secretase “a disintegrin and metalloproteinase” 10 (*Adam10*), which cleaves amyloid precursor protein (APP) in the non-amyloidogenic pathway [19]. The overexpression of PPARα (via lentivirus) increased the expression of ADAM10 in *Pparα* knockout neurons [19]. Furthermore, using gemfibrozil (an agonist of PPARα), PPARα:RXRα was recruited to the *Adam10* promoter in mouse hippocampal neurons and reduced β-amyloid (Aβ) production [19]. Given our understanding of the ability of ADAM10 to alleviate the burden of Aβ in AD [20,21,22], the therapeutic role of PPARα activation in AD should also be considered. Another study showed that peroxisomal proliferation by Wy-14,643 (a selective agonist of PPARα with weak agonists of PPARγ and PPARδ; EC_50_ values: 0.63, 32, and >100 μM at PPARα, PPARγ, and PPARδ, respectively) increased PPARα expression and attenuated Aβ-dependent toxicity in primary rat hippocampal neurons [23]. In a double transgenic mouse model of AD co-expressing a mutant human amyloid-β protein precursor (AβPPswe) and presenilin 1 without exon 9 (PS1dE9), 4-phenylbutyrate and Wy-14,643 (two agonists of PPARα) reduced spatial memory loss and Aβ neuropathology, prevented tau phosphorylation (known to induce the formation of neurofibrillary tangles and neuropil threads during the progression of AD [24,25]), and mitigated the loss of the synaptic protein [26]. Fenofibrate (an agonist of PPARα) also showed therapeutic effects on the amyloidogenic processing of APP through the PPARα/PI3K pathway in a transgenic mouse model of AD, which overexpresses APP/PS1 [27]. Pterostilbene, another agonist of PPARα, improved performance in spatial learning and memory tasks tested by a radial arm water maze in SAMP8 mice (a model of sporadic and age-related AD) and rescued a reduction in PPARα expression in the hippocampus of SAMP8 mice [28]. Although further investigation is needed, these data suggest that PPARα activation in the brain could moderate the progression of AD.

In the disease state of neuropsychiatric disorders, PPARα modulation has also been suggested as a novel therapeutic target [29]. Wy-14,643 showed anti-depressant effects in the forced swim test, tail suspension test, and chronic social defeat stress conditions in mice via the promotion of the BDNF signaling pathway [30]. As BNDF is a key determinant of anti-depressant effects [31], mood regulation through PPARα activation could be promising for the treatment of neuropsychiatric disorders. Based on next-generation sequencing (NGS) analysis, c.209-2delA, His117Gln, Arg141Cys, and Arg226Trp of the PPARA gene were found to be risk variants for schizophrenia in 1200 Japanese patients with schizophrenia [29]. Furthermore, behavioral deficits and impaired synaptogenesis in the cerebral cortex similar to schizophrenia were seen in *Pparα* knockout mice [29]. Treatment with fenofibrate alleviated spine pathology caused by phencyclidine (a schizophrenia-mimetic agent, one of NMDA receptor agonists) and reduced sensitivity to MK-801 (a hallucinogenic agent, one of NMDA receptor agonists) [29]. In other neuropsychiatric disorders such as post-traumatic stress disorder (PTSD) and major depressive disorders, PPARα activation by *N*-palmitoylethanolamine (PEA, an agonist of PPARα) improved contextual fear responses, facilitated fear extinction, and induced anxiolytic effects under a socially isolated condition in mice [32]. PEA has also been examined for neuroprotective effects in a murine model of Parkinson’s disease (PD) induced by treatment with 1-methyl-4-phenyl-1,2,3,6-tetrahyropyridine (MPTP), which destroys dopaminergic neurons in the substantia nigra [33]. Specifically, pathological microglial and astrocytic activation as well as damages in microtubule-associated protein, dopamine transporter, and nNOS expressions in the substantia nigra were lessened after treatment with PEA [33]. PEA treatment also decreased MPTP-associated behavioral impairments and motor deficits [33]. Finally, PEA-induced neuroprotection was found to be partially PPARα-dependent through experiments in *Pparα* knockout mice [33]. Another PPARα agonist (fenofibrate) also showed similar therapeutic effects on the progression of PD by preventing MPTP-induced cell death in the substantia nigra [34].

Similar to other neurodegenerative diseases, neuroinflammation is considered an important contributor to the progression of amyotrophic lateral sclerosis (ALS) [35,36]. The brain (especially the motor cortex and brainstem), spinal cord, and skeletal muscles innervated by neurons are all affected in ALS [35,36]. Continuous systemic administrations of fenofibrate improved ALS-like phenotypes, such as weight loss and motor dysfunction analyzed by rotarod testing, and extended the survival rates in SOD1^G93A^ mice (a widely used preclinical model for ALS [37]) [38]. Attenuation in spinal neuronal cell loss, as well as spinal cord gliosis, was observed in SOD1^G93A^ mice treated with fenofibrate [38]. An induction of PPARα expression and reductions in the expression of inflammatory molecules such as iNOS and COX-2 were suggested as the therapeutic reasons behind these observations [38].

Multiple sclerosis (MS) is another immune disease (an autoimmune disease) in the CNS (the brain and spinal cord) [39]. Autoreactive T cells migrate to the CNS and evoke severe inflammatory processes, particularly demyelinating events in the CNS [40]. These abnormal events lead to an axonal loss in CNS neurons, finally resulting in physical, neurological, and psychiatric problems [39,41]. Since PPARα activation could regulate inflammation, PPARα agonists have also been examined as potential therapeutics for MS [42,43]. Experimental autoimmune encephalomyelitis (EAE) was induced to develop a common experimental model of MS [44]. Treatment with gemfibrozil and fenofibrate inhibited EAE clinical signs in mice [42]. PPARα agonists (gemfibrozil and fenofibrate) had suppressive effects on CD4-positive Ag-specific proliferation [42]. Furthermore, gemfibrozil treatment could shift the cytokine secretion of T cell lines through the inhibition of IFN-γ production and the promotion of IL-4 production [42]. This implies that PPARα activation could modulate immune responses in MS. A previous report demonstrated greater PPARα expression in male CD4-positive cells than female CD4-positive cells. This difference was associated with reductions in NF-κB and c-Jun activities as well as an induction in IFN-γ levels [45]. Male *Pparα* knockout mice showed more severe clinical signs than female *Pparα* knockout mice after induction of EAE [45]. Based on this outcome, this group suggested that the abundant T cell expression of PPARα may be one of the factors driving males to be less prone to develop Th1-mediated autoimmunity than females in MS [46]. Fenofibrate also showed modulatory effects of other immune responses [47,48]. Lipopolysaccharide (LPS)-induced IL-12 family protein expressions were suppressed by fenofibrate treatment in primary mouse microglia and astrocytes [47]. Furthermore, fenofibrate treatment inhibited mRNA expressions of IL-12 family subunits in EAE mice [47]. As IL-12 family proteins have been known to play a crucial role in the generation of Th1 cells and the development of autoimmune diseases [49,50,51,52], the therapeutic effects of fenofibrate could be promising in MS.

In the disease state of ischemic stroke, PPARα activation has also been suggested as a therapeutic target. Fenofibrate treatment during the acute phase of experimental stroke in rats by transient middle cerebral artery occlusion (MCAO) in combination with thrombolysis by tissue plasminogen activator (tPA) exerted a reduction in the infarction volume (total, cortical, and striatal areas) and increased expression of ICAM-1 (a marker of leukocyte/endothelium interactions) and CD11b (a marker of activated microglia) [53]. Another study showed that fenofibrate treatment improved cerebral blood flow (CBF) in a murine model of ischemic stroke by transient MCAO [54]. However, fenofibrate treatment did not improve CBF in *Pparα*-null mice [54]. Similar therapeutic effects were seen in the exogenous administration of PEA in a murine model of ischemic stroke by transient MCAO, including a reduction in the infarction volume, astrocytic activation, and increased expressions of pro-inflammatory markers [55,56]. These outcomes imply that PPARα modulation may play a critical role in cerebrovascular protection in the ischemic brain. Taken together, reports on the therapeutic roles of PPARα activation in various brain diseases are growing in number (Figure 2).

## 3. Spinal Cord Diseases

Although less work has described the therapeutic potential of PPARα activation in spinal cord injuries, beneficial effects of PPARα activation have been reported. The expression of PPARα was detected in the rat spinal cord [57,58]. After subcutaneous injection of complete Freund’s adjuvant (CFA) to a hind paw in rats, the PPARα isotype was activated rapidly only in the rat spinal cord [59]. Even though we could not conclude any role of PPARα activation during hyperalgesia with these observations, PPARα could be considered responsive to pain pathways in the spinal cord. Using melatonin, which is the secretory immunomodulatory product of the pineal gland, the role of PPARα was examined in a mouse model of spinal cord trauma by vascular clipping to the dura in the spinal cord. Melatonin-mediated anti-inflammatory effects (suppression in infiltration of neutrophils, induction of pro-inflammatory cytokine, and activation of NF-κB) were weakened in *Pparα* knockout mice compared to those in wild-type mice [16]. Furthermore, Esposito et al. reported that PPARα might contribute to the anti-inflammatory activity of simvastatin (an inhibitor of 3-hydroxy-3-methylglutaryl coenzyme A reductase) in spinal cord injury [60]. The same group demonstrated that PPARα activation could contribute to anti-inflammation in spinal cord injuries using glucocorticoids (anti-inflammatory agents commonly used in the treatment of spinal cord trauma) in the same model of spinal cord trauma by vascular clipping to the dura in the spinal cord [61]. In summary, the anti-inflammatory effects of various drugs in spinal cord injuries were mediated by spinal PPARα activation. Conversely, a study using gemfibrozil (an FDA-approved drug for hyperlipidemia/an agonist of PPARα) exhibited opposing outcomes in spinal cord injured mice [62]. Locomotor dysfunction and histological impairments were exacerbated by treatment with gemfibrozil [62]. Therefore, we think that more investigations are needed to understand the therapeutic potential of PPARα activation in spinal cord injuries (Figure 2).

## 4. Eye Diseases

PPARα expression was considerably detected in the retina, cornea, and retinal pigment epithelium (RPE) of humans and mice [63,64,65,66]. The roles played by PPARα in maintaining homeostasis in the eye, including retinal protection, neovascularization, and retinal inflammation, have been well established. Fenofibrate Intervention in Event Lowering in Diabetes (FIELD) and Action to Control Cardiovascular Risk in Diabetes (ACCORD) are some of the largest clinical trials that focus on the role of fenofibrate in diabetic mellitus patients and complications such as diabetic retinopathy (DR) [67,68]. Based on these two clinical results, fenofibrate treatment could reduce the need for laser photocoagulation in patients with pre-existing retinopathy and slow the progression of DR [67,68]. Numerous experimental model studies have focused on explaining the therapeutic role of fenofibrate in DR. Streptozotocin-induced diabetic rats, and Akita mice (type 1 diabetes mellitus by a spontaneous point mutation in the *Ins2* gene), showed increased permeability in the retina, and its vascular leakage was reduced by the oral administration of fenofibrate [69]. Retinal vascular leukostasis was also decreased through treatment with fenofibrate in streptozotocin-induced diabetic rats [69]. When it comes to the modulation of retinal neovascularization, the intraocular delivery of fenofibrate could reduce the number of preretinal vascular cells in a rat model of oxygen-induced retinopathy, along with a reduction in vascular endothelial growth factor (VEGF) and hypoxia-inducible factor (HIF)-1α immunoreactivities in the retina [69]. Furthermore, PPARα-dependent therapeutic effects of fenofibrate on DR were confirmed using *Pparα* knockout animals [69]. The same group further demonstrated the neuroprotective effects of PPARα activation in the retinopathy of type 1 diabetes mellitus [70]. The oral administration of fenofibrate protected against visual dysfunction (analyzed by spatial frequency threshold), and intraperitoneal injection of fenofibric acid (a PPARα activator) reduced retinal apoptosis (analyzed by DNA fragmentation assay) [70]. Furthermore, using in vitro R28 cells (immortalized rat retinal precursor cells), the restoration of mitochondrial respiration by PPARα activation was confirmed under 4-hydroxynonenal (4-HNE)-induced oxidative stress condition [70]. As pericyte loss has been reported to occur in the early stage of DR and plays a critical role in its progression [71,72,73], the protective roles of PPARα were also examined in capillary pericytes in the diabetic retina by the same group [74]. Specifically, the administration of fenofibrate ameliorated the formation of retinal acellular capillary and loss of pericytes in a mouse model of streptozotocin-induced diabetes [74]. In *Pparα* knockout diabetic mice, the retinal acellular capillary was more severely formed [74]. A reduction in oxidative stress-induced apoptosis and reactive oxygen species production was observed by the activation and expression of PPARα in cultured primary human retinal capillary pericytes [74]. Furthermore, primary retinal pericytes obtained from *Pparα* knockout mice showed increased apoptosis under the same oxidative stress. Taken together, the therapeutic effects of PPARα activation in the diabetic retina have been identified using fenofibrate (Figure 2).

The study of the therapeutic effects of PPARα activation in the diabetic retina has continued using a new selective PPARα modulator (SPPARMα), pemafibrate [71]. Pemafibrate was designed to have higher potency and selectivity for PPARα activation than fenofibrate [75,76,77,78]. In this regard, pemafibrate showed fewer side effects on kidney injuries than fenofibrate [75,76,77,79]. As the safety concern regarding deleterious effects on renal function was raised in preclinical and clinical studies of fenofibrate [75,76,77,79], the use of pemafibrate became more promising in DR with renal dysfunction. Based on DNA microarray analysis and ChIP-seq of PPARα in human umbilical vein endothelial cells (HUVECs) incubated with pemafibrate, *THBD* expression (which encodes thrombomodulin) could be regulated by PPARα through its binding to the promoter region of THBD [80]. As thrombomodulin (one of the integral membrane proteins expressed in endothelial cells) has an important role in anti-inflammation [81,82,83], severe inflammation in DR could be modulated by PPARα activation (using pemafibrate) in the diabetic retina. The oral administration of pemafibrate inhibited VCAM-1 and MCP1 expression (inflammatory markers) in the rat streptozotocin-induced diabetic retina [80]. Knockdown of thrombomodulin by small interfering RNA attenuated the pemafibrate-mediated inhibition in VCAM-1 and MCP1 expression in the rat streptozotocin-induced diabetic retina [80]. Finally, the therapeutic effects of PPARα activation by pemafibrate on inhibiting retinal vascular leukostasis and leakage were mediated through the upregulation of THBD [80]. As excess extracellular glutamate is involved in retinal cell death in DR [84,85,86], the therapeutic effects of pemafibrate on retinal protection against DR were indirectly examined in a rat model of N-methyl-D-aspartate (NMDA)-induced excitotoxicity [87]. Treatment with pemafibrate reduced retinal ganglion cell loss induced by intravitreal injection of NMDA, and its protection was associated with the inhibition of c-Jun phosphorylation [87], which is linked to the induction of cell death-related genes [88]. In our previous study, the oral administration of pemafibrate exerted retinal protective effects in a murine model of DR by intraperitoneal injection of streptozotocin [89]. However, the therapeutic effects were not explained by ocular PPARα activation, as there was no significant change in PPARα target gene expressions by pemafibrate [89]. Rather, PPARα activation in the liver (analyzed by increases in PPARα target gene expressions including fibroblast growth factor 21; *Fgf21*) and induction of FGF21 in the serum, as well as improvements of blood glucose and lipid metabolisms, were suggested as drivers of the therapeutic effects of pemafibrate [89]. Similar effects were observed in a murine model of oxygen-induced retinopathy, where retinal neovascularization was suppressed by the oral administration of pemafibrate [90]. FGF21 is a hormone secreted by the liver [91] and has been reported to have suppressive effects on ocular neovascularization and vascular leakage in several animal models [92,93]. In this regard, therapeutic effects of pemafibrate on ocular neovascularization may be related to hepatic and systemic FGF21 induction. A recent report also demonstrated that fenofibrate reduced the severity of retinopathy in *db/db* mice (another mouse model of DR) without inducing PPARα-dependent gene expressions in the retina [94]. Rather, strong activation of PPARα in the liver was observed [94]. Taken together, further investigations on where PPARα activation exerts its therapeutic effects on DR are needed (Figure 2).

In the disease state of age-related macular degeneration (AMD), PPARα activation has also been suggested as a promising therapeutic target. Treatment with fenofibric acid reduced choroidal neovascularization (CNV) in a rat model of AMD by laser irradiation to the eye [95]. Its effects were explained by the downregulation in VEGF, TNF-α, and ICAM-1 expressions [95]. Furthermore, CNV was more developed in *Pparα* knockout mice than in wild-type mice [95]. As expected, the therapeutic roles of fenofibric acid on CNV were abolished in *Pparα* knockout mice [95]. As subretinal fibrosis and disruption of retinal iron homeostasis are also pathological outcomes in AMD, the therapeutic effects of fenofibrate were examined in relation to these aspects [96,97]. Fenofibrate inhibited subretinal fibrosis in the retina of very low-density lipoprotein receptor (*Vldlr*) knockout mice, which is one of the models of AMD for subretinal fibrosis [96]. Fenofibrate treatment inhibited two fibrotic signaling pathways (TGF-β-Smad2/3 and Wnt) in the *Vldlr* knockout retina [96]. An additional study demonstrated that fenofibrate treatment prevented iron-induced activation of oxidative stress and Wnt/β-catenin signaling in the eye [97]. As oxidative stress-induced injuries to RPE are implicated in the progression of AMD [98,99], therapeutic roles of PPARα activation were directly tested in adult retinal pigment epithelial cell line-19 (ARPE-19) using sulindac (one of the first nonsteroidal anti-inflammatory drugs) [100]. Sulindac protection against oxidative stress-induced RPE damages by tert-butylhydroperoxide (TBHP) or UVB light exposure was found to be PPARα-dependent [100]. Taken together, PPARα activation could aid in slowing the progression of AMD (Figure 2).

In the disease state of an ocular ischemic syndrome (OIS), little is known about the therapeutic roles of PPARα activation. Nonetheless, based on our recent studies, fenofibrate and pemafibrate showed neuroprotective effects (analyzed by electroretinography) via boosting liver PPARα function with systemic induction of FGF21, which is one of the neuroprotective molecules in the CNS [101,102]. Furthermore, pemafibrate treatment exerted the modulation of pathological gliosis in the ischemic retina to reduce ischemic damages in the inner retina [102]. Although the functions of PPARα were only examined in the liver and retina, we suspect that PPARα activation by pemafibrate/fenofibrate may not be limited to the liver. A recent report demonstrated that fenofibrate treatment increased circulating hematopoietic stem cells (possibly from the bone marrow) [103]. As OIS is closely related to circulation abnormalities in cardiovascular diseases, more comprehensive investigations of PPARα activation by pemafibrate/fenofibrate are necessary (Figure 2).

In the disease state of corneal diseases, the therapeutic roles of PPARα activation have been studied. In the streptozotocin-induced diabetic rat cornea and diabetic human cornea, a decrease in PPARα expression was detected [104], implying that the functions of PPARα in the cornea could be impaired by diabetes. Fenofibrate treatment reduced a loss of corneal nerve fiber density in streptozotocin-induced diabetic rats [104]. In mice, *Pparα* knockout showed decreases in corneal nerve fiber density and corneal sensitivity and an increase in the incidence of corneal lesions at the chronic stage [104]. These data suggest that targeting PPARα may potentially protect against corneal degeneration induced by diabetes and/or aging. The suppression of corneal neovascularization has been suggested as an additional therapeutic effect of PPARα activation in the cornea. Fenofibrate treatment suppressed corneal neovascularization by reducing *Vegf* and *Ang-2* mRNA expressions in a rat corneal alkali burn model [105]. The same group demonstrated that treatment with a mixture of fenofibrate/pioglitazone (combination of PPARα and PPARγ activation) also suppressed corneal neovascularization by reducing *Vegf* and *Ang-2* mRNA expressions in a rat alkali burn model [106]. Another group showed that the oral administration of PPARα agonists (fenofibrate, WY14,643, ETYA, bezafibrate, and gemfibrozil) suppressed FGF2-induced corneal neovascularization [107]. Taken together, the therapeutic roles of PPARα activation in corneal diseases have been established (Figure 2).

## 5. Future Perspectives

**Figure 3:**
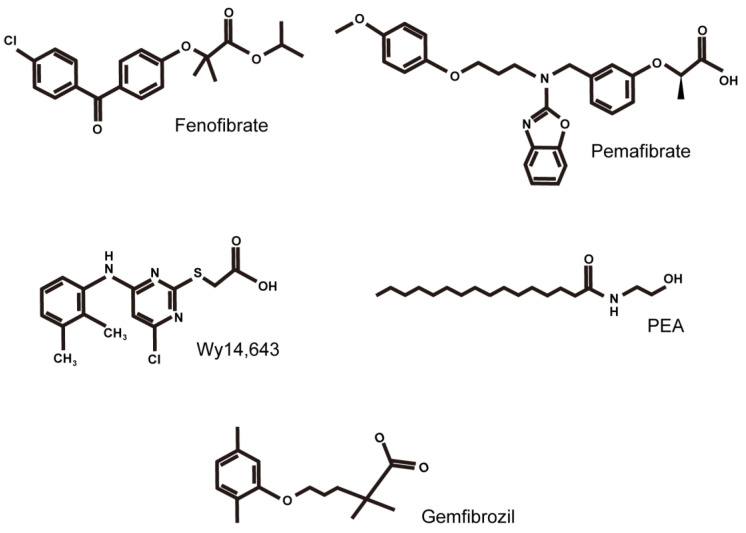
Structural formulas of PPARα agonists are mainly discussed in this review article. Formulas of fenofibrate, pemafibrate, Wy14,643, PEA (*N*-palmitoylethanolamine), and gemfibrozil are listed from the upper left to the bottom right.

Thus far, when reviewing the therapeutic roles of PPARs in the CNS, PPARγ has received the most attention, as it shows promising effects against CNS diseases. As a result, the role of PPARα has been less discussed. In this review article, we summarized recent reports of PPARα modulation therapy with agonists of PPARα (Figure 3) in CNS diseases, from the brain to the eye in an attempt to generate a more comprehensive understanding of the protective roles of PPARα in CNS diseases. Although more investigations on the therapeutic roles of PPARα in CNS diseases are needed, we think that PPARα and PPARγ share a number of neurophysiological roles, which include the regulation of neuroinflammation, neuroprotection, and stress responses, and the modulation of cognition, anxiety, and emotional actions in the CNS [108,109,110,111]. On the other hand, understanding the therapeutic roles of PPAR-δ/β in CNS diseases is limited, as it was discovered later, and less research has been conducted. Thus, a comprehensive summarization of the role of PPAR-δ/β in CNS diseases requires more time and effort.

The CNS maintains unique and important physiological barriers from the peripheral circulation, termed “the blood–brain barrier” and/or “the blood–retina barrier”. For CNS drug delivery, more research is needed on how to effectively deliver PPAR agonists to the CNS or activate PPARs locally in various CNS tissues. Along with our current summary (Figure 2), we urge further research to obtain more solid evidence on PPARα modulation therapy in CNS diseases.

## Data Availability

Not applicable.
